# Influence of obesity in pulmonary function and exercise tolerance in obese women with obstructive sleep apnea

**DOI:** 10.20945/2359-3997000000101

**Published:** 2019-02-01

**Authors:** Vivian Maria Moraes Passos, Anna Myrna Jaguaribe de Lima, Bárbara Renatha Afonso Ferreira de Barros Leite, Rodrigo Pinto Pedrosa, Isly Maria Lucena de Barros, Laura Olinda Bregieiro Fernandes Costa, Amilton da Cruz Santos, Maria do Socorro Brasileiro-Santos

**Affiliations:** 1 Universidade de Pernambuco Universidade de Pernambuco Centro Integrado de Saúde Amaury de Medeiros Recife PE Brasil Centro Integrado de Saúde Amaury de Medeiros, Universidade de Pernambuco (UPE), Recife, PE, Brasil; 2 Universidade Federal Rural de Pernambuco Universidade Federal Rural de Pernambuco Departamento de Morfologia e Fisiologia Animal Recife PE Brasil Departamento de Morfologia e Fisiologia Animal, Universidade Federal Rural de Pernambuco (UFRPE), Recife, PE, Brasil; 3 Pronto-Socorro Cardiológico de Pernambuco Pronto-Socorro Cardiológico de Pernambuco Laboratório do Sono e Coração Recife PE Brasil Laboratório do Sono e Coração, Pronto-Socorro Cardiológico de Pernambuco (PROCAPE), Recife, PE, Brasil; 4 Pronto-Socorro Cardiológico de Pernambuco Pronto-Socorro Cardiológico de Pernambuco Departamento de Cardiologia Recife PE Brasil Departamento de Cardiologia, Pronto-Socorro Cardiológico de Pernambuco (PROCAPE), Recife, PE, Brasil; 5 Universidade de Pernambuco Universidade de Pernambuco Departamento de Ginecologia e Obstetrícia Recife PE Brasil Departamento de Ginecologia e Obstetrícia, Universidade de Pernambuco (UPE), Recife, PE, Brasil; 6 Universidade Federal da Paraíba Universidade Federal da Paraíba Departamento de Educação Física João Pessoa PB Brasil Departamento de Educação Física, Universidade Federal da Paraíba (UFPB), João Pessoa, PB, Brasil

**Keywords:** Obesity, obstructive sleep apnea, lung function, exercise tolerance

## Abstract

**Objective:**

To evaluate the influence of obesity on pulmonary function and exercise tolerance in women with obstructive sleep apnea (OSA).

**Subjects and methods:**

A descriptive analytic cross- sectional study was carried out. Thirty-nine (39) sedentary climacteric women, aged 45 to 60 years, were evaluated and submitted to polysomnography. The participants were divided into 4 groups: a) ‘eutrophic non-OSA’ (n = 13); b) ‘eutrophic OSA’ (n = 5); c) ‘obese non-OSA’ (n = 6); d) ‘obese OSA’ (n = 15). All subjects underwent clinical and anthropometric evaluation, followed by pulmonary function tests and 6-minute walk test (6MWT).

**Results:**

There was a significant difference in the predicted percentage values of FEV_1_/FVC when comparing ‘eutrophic OSA’ and ‘obese OSA’ (97.6% ± 6.1% vs. 105.7% ± 5.7%, respectively; p = 0.025). The other spirometric variables did not show any differences between the studied groups. There was no significant difference in the maximum distance walked when the ‘eutrophic non-OSA’, ‘eutrophic OSA’, ‘obese non-OSA’ and ‘obese OSA’ groups were compared.

**Conclusion:**

Considering the results of this study, OSA itself did not influence pulmonary function or functional capacity parameters compared to eutrophic women. However, not only isolated obesity but also obesity associated with OSA can negatively impact sleep quality and lung function.

## INTRODUCTION

Obstructive sleep apnea (OSA) is a respiratory disorder characterized by repeated and cyclic episodes of total collapse of the upper airway during sleep. These events may result in oxygen desaturation, high blood levels of carbon dioxide and microarousals (1,2). Recent studies have estimated that nearly 4% of men and 2% of women in the world, aged between 30 to 60 years, suffer from OSA (3). Tufik and cols. (4) showed that in São Paulo, Brazil's most populous city, approximately 32% of the population present risk factors for any degree of OSA severity, and its prevalence increases with age for men and women, although it is higher in males.

There is also a high prevalence of OSA in obese individuals, mainly due to fat accumulation in the neck region, morphological modification of upper airways and lung volume reduction (2,3,5). Women of working/reproductive age tend to have less OSA than men, possibly as a result of protective action of progesterone over the pharyngeal dilator muscles. Leptin levels seem to have an influence on ventilatory control, promoting better responses to hypoxia and hypercapnia (6). In contrast, post-menopausal women have higher prevalence and severity of OSA caused by the loss of hormonal protection (3,6,7).

Regarding the deleterious effects of obesity on pulmonary function, reduced forced expiratory volume in the first second (FEV_1_) is observed, as well as reduced forced vital capacity (FVC) and their relation, lower strength and endurance of the respiratory muscles (5,8). In obese women, there is a reduction in the respiratory function, with lower vital capacity (VC) and diminished maximum voluntary ventilation (MVV) (9).

To our knowledge, this is the first paper that has studied exercise tolerance in obese women with OSA. Therefore, the aim of this study was to evaluate the influence of obesity on pulmonary function, cardiorespiratory fitness and sleep quality in women with obstructive sleep apnea.

## SUBJECTS AND METHODS

This is a cross-sectional descriptive study, approved by the Ethics and Research Committee of the Universidade Federal de Pernambuco. The participants were selected through the medical records of the patients admitted to the Heart and Sleep Laboratory – PROCAPE, based on their previous polysomnography exam.

Polysomnography was performed in the same laboratory, where all patients were monitored overnight using an Embletta-type handheld portable respirator (Embla, Embletta^®^ Gold, USA) (10). This test aimed to identify the Apnea-Hypopnea Index (AHI), which is defined by the mean number of apnea and hypopnea events per hour, in order to confirm diagnosis and to measure OSA severity according to definitions of the American Academy of Sleep Medicine (11): i) absence of OSA < 5 events/hour); ii) mild OSA (5 < AHI < 15 events/hour); iii) moderate OSA (15 < AHI < 30 events/hour); iv) severe OSA (AHI > 30 events/hour).

Women who were submitted to a polysomnography, aged between 45 and 60 years, eutrophic and grade I and II obese (12) were included in the study. Overweight and morbidly obese women, as well as smokers or people who had quit smoking for at least one year were excluded from the study. Participants receiving hormone replacement therapy or with respiratory disorders besides OSA, subjects who could not comprehend the verbal commands during evaluation or showed discomfort during the exams were excluded. Each participant provided written informed consent to participate.

Sample size calculation was performed using the GPower^®^ software, version 3.1.6 (Kiel University, Germany), using a probabilistic error of 0.05, effect size of 0.25, and 80% statistical power as parameters, so that the minimum sample was determined as a total of 20 subjects (n = 5 in each group).

Pittsburgh Sleep Quality Index. The Pittsburgh sleep quality index (PSQI), validated for Brazilian population (13), is a self-reported measure of sleep quality and queries about sleep-related variables over the previous month. The global score varies from 0 to 21, and higher results indicate poor sleep quality.

Physical Activity Questionnaire. The physical activity questionnaire (14), validated for the Brazilian population and remained from the original International Physical Activity Questionnaire (IPAQ), is also a self-report questionnaire to detect the level physical fitness.

Lung Function Evaluation. During spirometric tests (Spirobank G^®^ USB (MIR, Rome, Italy), the patients remained seated, back steady and performed the forced vital capacity (FVC) and maximum voluntary ventilation (MVV) maneuvers. In every test, the maneuvers should conform with the criteria of the American Thoracic Society (ATS) (15). The resulting measurements of all tested maneuvers were FVC, FEV_1_, FEV_1_/FVC ratio, forced expiratory flow between 25 and 75% (FEF25%-75%), peak expiratory flow (PEF) and the MVV. All the results were expressed as an absolute value and percentage of the predicted numbers (16).

Cardiorespiratory Fitness Evaluation. Six-minute walk test (6MWT), a submaximal effort test, was performed according to ATS (17) rules. Parameters such as saturation of peripheral oxygen (SpO2), heart rate (HR), respiratory rate (RR), systolic and diastolic blood pressure (SBP and DBP) and rate of perceived exertion were measured before and after the 6MWT, as recommended.

Statistical Analysis. Statistical analysis was performed using the Statistical Package for the Social Sciences-SPSS 15.0 (Statsofat Inc., Chicago, IL, USA, 2006). The Kolmogorov-Smirnov test evaluated normality of the data. ANOVA repeated measures was used to compare means within and between groups and post-hoc analysis was evaluated by Bonferroni test. Fischer's exact test verified differences between categorical variables. The results are presented as mean ± standard deviation. A p-value < 0.05 was considered statistically relevant for all analyses.

## RESULTS

Out of the 265 medical records evaluated in the Heart and Sleep Laboratory – PROCAPE, 178 provided information that did not match the inclusion criteria. From the 87 patients remaining, 21 were excluded because they did not fill the eligibility criteria when contacted and 66 were considered eligible. Then, 19 subjects refused to participate, and another 8 started to participate in the study but were unable to perform the necessary tests, comprising a total sample loss of 226 subjects.

The final sample consisted of 39 women, aged between 45 to 60 years, divided into the groups 1) ‘eutrophic non-OSA’ (n = 13), 2) ‘eutrophic OSA’ (n = 5), 3) ‘obese non-OSA’ (n = 6) and 4) ‘obese OSA’ (n = 15). In the ‘eutrophic OSA’ group, the prevalence of mild and moderate apnea was 80% and 20%, respectively.

In the ‘obese OSA’ group, the results were 40% for mild OSA, 47% for moderate OSA and 13% for severe OSA. Anthropometric measures are presented in Table 1. When comparing the eutrophic group with the obese group, regardless of OSA, a significant difference related to weight, body mass index (BMI), neck circumference and waist-hip ratio (WHR) were observed. Likewise, when comparing OSA and non-OSA groups, regardless of obesity, different AHI results were detected, as expected. When comparing the ‘eutrophic OSA’ group and the ‘obese OSA’ group, the latter had significantly higher AHI levels (8.94 vs. 17.98; p = 0.032) (Table 1).

**Table 1:** Sample characteristics, anthropometric information, apnea classification (AHI) and sleep quality (PSQI)

Variables	Groups				
	Eutrophic non-OSA (n = 13)	Eutrophic OSA (n = 5)	Obese non-OSA (n = 6)	Obese OSA (n = 15)
	Mean ± SD	Mean ± SD	Mean ± SD	Mean ± SD
Age (years)	52.5 ± 4.2	50.0 ± 12.1	50.0 ± 1.4	53.1 ± 6.22^f^
Weight (kg)	54.2 ± 6.18	53.5 ± 10.8	84.2 ± 10.72^b^.^d^	85.5 ± 10.02^c^.^e^
Height (m)	1.54 ± 0.05	1.55 ± 0.09	1.6 ± 0.07	1.6 ± 0.08^e^
BMI (kg/m^2^)	22.8 ± 2.0	22.1 ± 2.4	33.8 ± 3.09^b^.^d^	34.0 ± 1.79^c^.^e^
Neck Circumference (cm)	32.0 ± 1.8	33.1 ± 0.9	37.2 ± 1.3^b^.^d^	36.9 ± 2.59^c^.^e^
Waist (cm)	82.6 ± 7.9	78.4 ± 8.1	103.2 ± 8.8^b^.^d^	105.5 ± 6.83^c^.^e^
Hip (cm)	94.4 ± 5.6	94.3 ± 10.3	111.7 ± 6.5^b^.^d^	114.2 ± 5.58^c^.^e^
WHR	0.88 ± 0.08	0.83 ± 0.05	0.92 ± 0.04^d^	0.93 ± 0.06^e^
AHI (events/hour)	1.8 ± 1.5	8.9 ± 2.9^a^	2.3 ± 1.7^d^	18.0 ± 10.7^c^.^e^.^f^
PSQI	9.1 ± 3.0	9.8 ± 3.6	6.2 ± 2.5^b^,^d^	9.7 ± 3.5^f^

BMI: body mass index; WHR: waist hip ratio; AHI: apnea hypopnea index; PSQI: Pittsburgh sleep quality index.

a p < 0.05 Eutrophic non-OSA x Eutrophic OSA;

b p < 0.05 Eutrophic non-OSA x Obese non-OSA;

c p < 0.05 Eutrophic non-OSA x Obese OSA;

d p < 0.05 Eutrophic OSA x Obese non-OSA;

e p < 0.05 Eutrophic OSA x Obese OSA;

f p < 0.05 Obese non-OSA x Obese OSA.

Sleep quality assessed with PQSI did not differ between eutrophic groups (OSA or non-OSA) (9.80 ± 6.63 and 9.08 ± 3.04; p = 0.617). The ‘eutrophic OSA’ group had higher PQSI results than the ‘obese OSA’ (9.08 ± 3.04 vs. 6.17 ± 2.48, p = 0.01), as well as comparing the ‘eutrophic OSA’ and ‘obese non-OSA’ (9.80 ± 3.04 vs. 6.17 ± 2.48; p = 0.04). In comparing the obese OSA and non-OSA, there were also different results (9.73 ± 3.53 vs. 6.17 ± 2.48, respectively; p = 0.035) (Table 1). Groups were paired in percentage of non-smokers/ex-smokers, presence of dyslipidemia and diabetes mellitus (DM).

However, the presence of systemic arterial hypertension (SAH) was more expressive in the ‘obese OSA’ group (Table 2). Physical fitness obtained through IPAQ did not show differences between groups (p = 0.621) (Table 2).

**Table 2:** Sample characterization, tobacco smoking, comorbidities and physical activity level

Variables	Groups				p-value*
	Eutrophic non-OSA	Eutrophic OSA	Obese non-OSA	Obese OSA	
	n (%)	n (%)	n (%)	n (%)	
**Smoking**					
Non-smoker	10 (76.9)	5 (100.0)	4 (66.7)	10 (66.7)	0.585
Ex-smoker	3 (23.1)	0 (0.0)	2 (33.3)	5 (33.3)	
**SAH**					
Yes	7 (53.8)	0 (0.0)	4 (66.7)	12 (80.0)	0.016
No	6 (46.2)	5 (100.0)	2 (33.3)	3 (20.0)	
**Dyslipidemia**					
Yes	4 (30.8)	2 (40.0)	1 (16.7)	4 (26.7)	0.879
No	9 (69.2)	3 (60.0)	5 (83.3)	11 (73.3)	
**DM**					
Yes	1 (7.7)	0 (0.0)	1 (16.7)	4 (26.7)	0.532
No	12 (92.3)	5 (100.0)	5 (83.3)	11 (73.3)	
**IPAQ**					
MET (min/week)	273,0 ± 198,5	283,8 ± 148,1	256,9 ± 101,6	273,0 ± 198,5	0.621

SAH: systemic arterial hypertension; DM: diabetes mellitus; IPAQ: international physical activity questionnaire.

(*) Fisher's Exact Test.

There was only a significant difference in the predicted percentage values of FEV_1_/FVC when we compared eutrophic and obese OSA groups (97.60% ± 6.07% vs. 105.73% ± 5.73%, respectively; p = 0.025) (Table 3). The other spirometric variables did not show any differences between the studied groups. There was no significant difference in the maximum distance walked comparing the groups (Table 3).

**Table 3:** Pulmonary function test results and maximum distance walked in the 6MWT

Variables	Groups				
	Eutrophic Non-OSA (n = 13)	Eutrophic OSA (n = 5)	Obese non-OSA (n = 6)	Obese OSA (n = 15)
	Mean ± SD	Mean ± SD	Mean ± SD	Mean ± SD
FVC (l)	2.55 ± 0.45	3.05 ± 0.74	2.65 ± 0.64	2.60 ± 0.48
predicted FVC%	89.92 ± 16.44	102.00 ± 19.42	87.33 ± 19.65	86.47 ± 11.49
FEV_1_ (l)	2.16 ± 0.36	2.43 ± 0.60	2.26 ± 0.42	2.22 ± 0.40
predicted FEV_1_%	94.69 ± 16.36	98.60 ± 18.26	91.00 ± 15.28	92.60 ± 16.86
FEV_1_/FVC	84.92 ± 4.26	79.84 ± 6.16	86.38 ± 8.44	85.52 ± 4.87
predicted FEV_1_/FVC%	104.00 ± 5.52	97.60 ± 6.07	105.67 ± 9.61	105.73 ± 5.73^e^
PEF (l/s)	4.00 ± 0.90	3.91 ± 0.61	3.79 ± 0.43	4.33 ± 0.93
predicted PEF%	59.31 ± 13.33	56.80 ± 7.22	54.17 ± 4.88	62.13 ± 13.16
FEF_25-75%_ (l/s)	2.53 ± 0.41	2.47 ± 0.70	2.72 ± 0.46	2.82 ± 0.69
predicted FEF%	108.92 ± 19.94	99.20 ± 23.57	111.00 ± 17.74	119.07 ± 33.74
MVV (l/min)	82.74 ± 9.97	82.24 ± 12.68	90.87 ± 11.98	89.49 ± 19.18
predicted MVV%	75.69 ± 9.24	72.80 ± 8.96	75.67 ± 9.29	77.07 ± 15.81
Distance walked (m)	470.77 ± 8.22	462.72 ± 39.38	434.97 ± 51.32	453.11 ± 87.04

FEV_1_: forced expiratory volume in the first second; FVC: forced vital capacity; PFE: peak expiratory flow; FEV_1_/FVC: ratio of FEV_1_ to FVC; MVV: maximal voluntary ventilation.

e p < 0.05 Eutrophic OSA x Obese OSA.

## DISCUSSION

OSA did not seem to affect the pulmonary function of eutrophic and obese individuals in the present study, but in the latter group it caused poor sleep quality. On the other hand, the presence of obesity proved to be a condition that not only negatively interferes in sleep quality of subjects with or without OSA, but also in certain pulmonary function variables. However, exercise tolerance was not modified by any of these disorders in the evaluated population.

Regarding sleep quality, the PQSI had better results in the ‘eutrophic non-OSA’ when compared to the ‘obese non-OSA’, but there was no difference between the obese with OSA group. When validating the PQSI for the Brazilian population, Bertolazi and cols. (13) identified the results of this test from 2.5 ± 2.0 in control individuals to 8.1 ± 4.0 in subjects with OSA. In our sample, non-OSA participants showed different outcomes, suggesting that not only OSA but also other factors related to sleep may have interfered in the results.

In the groups with OSA, the results followed the ones observed in PSQI validation (13). Among obese individuals, the PQSI showed differences when compared to OSA or non-OSA subjects, pointing out that the presence of the disease decreases sleep quality, at least in these individuals. In fact, other studies using the Epworth sleepiness scale (ESS) showed that obese people with OSA have bad sleep quality when compared to control groups (18,19).

Regarding pulmonary function and OSA, it is very clear that there is a complex interaction between structure and function. Previous studies have shown a relation between OSA and reduced pulmonary volume such as residual volume, and a decline of expiratory reserve volume and vital capacity, with a reduction of airway patency which increases the possibility of collapse, thus leading to emergence or aggravation of OSA. However, these primary variables are not the purpose of our study. Our results revealed that the main spirometric variables were not influenced by OSA when eutrophic or obese groups with or without the disorder were compared, as previously observed (20). Regarding FEV_1_ and FVC, the negative relationship between obesity and pulmonary function has already been demonstrated (21,22). Previous studies have also shown that there is reduction in these parameters and the predicted percentage in individuals with severe OSA (23).

There were no differences in these two variables in the present study. It is possible that higher BMI as well as higher degrees of OSA may have interfered in the final results of those studies (22,23), which limits comparisons with our data. There were differences in the FEV_1_/FVC ratio in our study in terms of the predicted percentage between ‘eutrophic OSA’ and ‘obese OSA’, contradicting the results of other studies (21–23) which found that the quotient does not show a difference between eutrophic or obese groups with OSA or non-OSA due to simultaneous and proportional reduction of FEV_1_ and FVC. It is assumed that the severity of the OSA, initially different, may have affected some level of obstruction in the participants, promoting such results.

Regarding respiratory muscle resistance, Costa and cols. (9) showed that the MCC is reduced as the BMI increases. In the same way, Chien and cols. (23) observed a decrease of MVV in individuals with OSA. Their results are not compatible to our research, since our results did not show a difference between the studied groups. It is assumed that our MVV results were not influenced by OSA, or that the obese individuals may have received non-specific and indirect muscle training caused by the adipose tissue on the respiratory muscles, showing no differences in comparison to the eutrophic group. Data for FEF25-75% were similar in all the groups, corroborating the results of previous authors in which these variables do not change when comparing obese individuals with healthy control individuals (8,22). These answers explain the lower influence of obesity on expiratory volumes and confirm the restrictive character of it. However, there is evidence that this variable may decrease significantly, as long as there is a high BMI and presence of OSA (24).

With regards to cardiorespiratory fitness, our results did not show any difference in the maximum distance walked during 6MWT in any of the groups, assuming that there would be no interference from obesity or OSA in this parameter. When the eutrophic and obese groups were compared, there were no significant changes in the maximum distance walked due to the obesity factor. The results contradict studies performed with obese and eutrophic subjects, which showed that the latter walked a greater distance, and that the difference among women was bigger (25). These authors confirmed a negative and discrete relationship between obesity (and its severity levels) and the distance walked in the 6MWT.

Nevertheless, in comparing eutrophic and obese without OSA, Alameri and cols. (26) did not detect any differences in the distance walked, which supports the results of our research. Regarding the effects of OSA on cardiorespiratory fitness, just one previous study compared eutrophic individuals with or without OSA (27), and no difference was observed regarding VO_2_Max, respiratory exchange ratio (RER) or anaerobic threshold (AT) between the groups, which suggests no or little influence of the OSA on the functional capacity.

Other results reinforce the low influence that this respiratory disorder has on exercise tolerance, as there was no difference in variables such as VO2, RER and work rate by comparing groups with or without OSA or obese subjects separated by the level of OSA (28). Many previous studies have shown an association between OSA and reduced cardiorespiratory fitness. Some indicate that patients suffering with OSA have reduced functional capacity, since they observed lower VO_2_peak, VO_2_Max and AT values compared to the predicted values or in comparison to healthy individuals (29,30). In a gender comparison study, Cintra and cols. (31) showed that women with OSA in the climacteric period submitted to maximum effort test had lower oxygen consumption (VO_2_), reduced maximum heart rate (HRmax) and lower pressure levels than the ones found in men, which suggest that women with OSA have peculiarities during and after aerobic exercise.

Pływaczewski and cols. (19) also suggest that patients with severe OSA show exercise intolerance and point to the female gender, the presence of SAH, high BMI and low FVC as increased factors. Vanhecke and cols. (18) not only observed a reduction of VO_2_max in morbidly obese individuals with OSA when compared to individuals who do not suffer from the disorder, but also an inverse relation between this variable and AHI, even if the ventilatory equivalents remain similar between the groups.

Regarding the 6MWT, it was already seen that the distance walked was much higher in eutrophic with no OSA than in obese with OSA (26). It is important to note that the level of apnea was severe (average AHI of 66 events/hour) (26) for these authors, as well as the obesity level (average BMI of 50 kg/m^2^) (18), which may have boosted the outcomes.

The present study has some limitations. The low number of patients may have interfered in the results and some of the tested groups may have been even more negatively affected by this factor. The performance of this study was limited to one research center, what makes the data less extrapolated and compromises the generalization of the results. The results for pulmonary function could have been supplemented by evaluation of respiratory muscle strength, without which it is not possible to know if there is a muscular condition affecting the functional matter.

Regarding the submaximal functional capacity, one of the limitations was the fact that other factors that contribute to exercise tolerance were not investigated, such as peripheral muscular condition, heart function and psychological factors. Moreover, a maximum effort test could generate more reliable answers about the exercise tolerance for the studied population.

Considering the results of this study, OSA itself did not influence pulmonary function and functional capacity parameters when obese and non-obese women were compared, but this disorder improved poor sleep quality in obese subjects. However, not only isolated obesity but also obesity associated with OSA can negatively impact sleep quality and lung function. It is suggested that studies with a larger sample size with different grades of OSA and obesity, along with pulmonary strength tests and maximal cardiopulmonary exercise test and associations between lung function and anthropometric and polysomnographic variables should be considered to obtain more reliable answers about pulmonary function and exercise tolerance in obese women with OSA.
